# 7295 elderly hospitalized patients with catheter-associated urinary tract infection: a case-control study

**DOI:** 10.1186/s12879-023-08711-0

**Published:** 2023-11-24

**Authors:** Li Shen, Ting Fu, Luguang Huang, Huiying Sun, Yu Wang, Lili Sun, Xiaoyun Lu, Jing Zhang, Zhaoxu Yang, Chunping Ni

**Affiliations:** 1 Department of Disease Prevention and Control, The First Affiliated Hospital of Air Force Medical University, Changle West Road, Xincheng District, Xi’an, Shaanxi China; 2 Department of Military Prevention Medicine, Air Force Medical University, Changle West Road, Xincheng District, Xi’an, Shaanxi China; 3 Department of information, The First Affiliated Hospital of Air Force Medical University, Changle West Road, Xincheng District, Xi’an, Shaanxi China; 4 Neurosurgical ICU, The First Affiliated Hospital of Air Force Medical University, Changle West Road, Xincheng District, Xi’an, Shaanxi China; 5 Neurological ICU, The First Affiliated Hospital of Air Force Medical University, Changle West Road, Xincheng District, Xi’an, Shaanxi China; 6 Department of Nursing, Air Force Medical University, Changle West Road, Xincheng District, Xi’an, Shaanxi China

**Keywords:** Elderly, Catheter-associated urinary tract Infection, Risk factors, Pathogen distribution, Clinical characteristics, Outcomes

## Abstract

**Background:**

Catheter-associated urinary tract infection (CAUTI) ranks second among nosocomial infections in elderly patients after lung infections. Improper treatment can lead to death. This study analysed the risk factors, pathogen distribution, clinical characteristics and outcomes of CAUTI in elderly inpatients with a large sample size to provide evidence for clinical prevention and control.

**Methods:**

Based on the HIS and LIS, a case‒control study was conducted on all hospitalized patients with indwelling urinary catheters ≥ 60 years old from January 1, 2019, to December 31, 2022, and the patients were divided into the CAUTI group and the non-CAUTI group.

**Results:**

CAUTI occurred in 182 of 7295 patients, and the infection rate was 3.4/per 1000 catheter days. Urine pH ≥ 6.5, moderate dependence or severe dependence in the classification of self-care ability, age ≥ 74 years, male sex, hospitalization ≥ 14 days, indwelling urinary catheter ≥ 10 days, diabetes and malnutrition were independent risk factors for CAUTI (*P* < 0.05). A total of 276 strains of pathogenic bacteria were detected in urine samples of 182 CAUTI patients at different times during hospitalization. The main pathogens were gram-negative bacteria (n = 132, 47.83%), followed by gram-positive bacteria (n = 91, 32.97%) and fungi (n = 53, 19.20%). Fever, abnormal procalcitonin, positive urinary nitrite and abnormal urination function were the clinical characteristics of elderly CAUTI patients (*P* < 0.001). Once CAUTI occurred in elderly patients, the hospitalization days were increased by 18 days, the total hospitalization cost increased by ¥18,000, and discharge all-cause mortality increased by 2.314 times (*P*<0.001).

**Conclusion:**

The situation of CAUTI in the elderly is not optimistic, it is easy to have a one-person multi-pathogen infection, and the proportion of fungi infection is not low. Urine pH ≥ 6.5, moderate or severe dependence on others and malnutrition were rare risk factors for elderly CAUTI in previous studies. Our study analysed the clinical characteristics of CAUTI in the elderly through a large sample size, which provided a reliable basis for its diagnosis and identified the adverse outcome of CAUTI.

## Background

Urinary catheterization assisted by an indwelling urinary catheter is essential in treating most diseases. It not only brings clinical convenience but also becomes a significant risk factor for urinary tract infection (UTI). The aging of the global population is developing rapidly [1]. The problem of nosocomial infection in elderly patients has received increasing attention. Several studies have shown that advanced age is a significant risk factor for catheter-associated urinary tract infection (CAUTI) [2–4].Improper treatment can lead to death, and the case fatality rate is approximately 6.2% [5].

Approximately 20% of patients need indwelling urinary catheters during hospitalization, especially elderly patients [6]. In the UK, there are approximately 90,000 long-term catheter users [7]. Surveys from 11 European countries show that 5.4% of people over 65 need long-term indwelling urinary catheters [8]. However, the immunity of elderly patients decreases with age, and coupled with the gradual decline in organ function, the risk of related infection will increase. In fact, most elderly and stroke patients have a long-term bedridden status, further decline in immunity and so on. In this case, UTIs occur easily.

Although the incidence of CAUTI is high, accounting for approximately 40% [9] of nosocomial infections, 65-70% of CAUTIs are preventable [10]. The existing prevention and control measures are still based on early extubation and reduction of unnecessary indwelling catheters [11]. Nevertheless, it is not necessarily applicable based on the disease characteristics of elderly patients. The most critical risk factors for CAUTI in elderly patients are a long indwelling urinary catheter and a long hospitalization time. This is usually because the condition is severe and takes a long time to recover. Therefore, we urgently need to carry out a large sample of high-quality clinical research for elderly patients to seek effective prevention and control measures for elderly patients who need an indwelling urinary catheter.

## Methods

### Study design and participants

All patients were ≥ 60 years old with indwelling urinary catheters during hospitalization from January 1, 2019, to December 31, 2022. Inclusion criteria: ① Patients ≥ 60 years old; ② Patients with indwelling urinary catheter>2 days (calendar days). Exclusion criteria: ① Confirmed UTI or indwelling urinary catheter before admission; ② No routine urine test was performed before indwelling urinary catheter was placed in this hospital. ③ This hospitalization was related to urinary system diseases; ④ Research variables and indicator-related information were incomplete; ⑤ Patients with acidosis, alkalosis and hyperuricemia were diagnosed before or during hospitalization; ⑥ Military retirees or patients with continuous hospitalization for more than one year due to medical disputes. This study was approved by the Ethics Committee of the First Affiliated Hospital of Air Force Medical University (XJLL-KY-20,232,139).

Obtainment of informed consent from each participant was waived by the Ethics Committee of the First Affiliated Hospital of Air Force Medical University due to the nature of the study. All methods were performed following the Ethical Review of Biomedical Research Involving Human Beings issued by the National Health and Family Planning Commission of China.

### Diagnostic criteria

The diagnosis of CAUTI was carried out according to the American CDC/NHSN [12]: 1.The patient had an indwelling urinary catheter that had been in place for > 2 days on the date of the event (day of device placement = Day 1) and was either still present on the date of the event or removed the day before the date of the event. 2. The patient had at least one of the following signs or symptoms: (1) fever (> 38.0 °C).(2) suprapubic tenderness. (3)costovertebral angle pain or tenderness. 3.The patient had a urine culture with no more than two species of organisms,at least one of which was a bacteria of ≥ 10^5^ CFU/ml. If more than two types of microorganisms were isolated, the sample was considered to be contaminated.

The CAUTI rate per 1000 urinary catheter days was calculated by dividing the number of CAUTIs by the number of catheter days and multiplying the result by 1000.

### Etiological examination

The quality control strains *Staphylococcus aureus*(*S.aureus*) (ATCC29213), *Escherichia coli* (*E.coli*)(ATCC25922), *Klebsiella pneumoniae*(*K.pneumoniae*) (ATCC700603), and *Pseudomonas aeruginosa*(*P.aeruginosa*) (ATCC27853) were all purchased from the Clinical Laboratory Center of the National Health Commission. Urine samples were collected from suspected CAUTI patients and sent to the clinical laboratory within 2 h. After that, the specimens were inoculated into the corresponding media(sheep blood agar medium was used for bacteria, and Sabourand agar medium was used for fungi) and identified using a VITEKMS microbial mass spectrometer (BioMérieux, France) according to the fourth edition of the National Clinical Laboratory Procedures.

The same pathogen detected in the urine samples of the same patient at different times was counted as only one strain.

### Statistical analysis

SPSS 26.0 was used for statistical analysis. Counting data are expressed in frequency and percentage terms, and measurement data are described by the mean ± standard deviation or median (interquartile distance) according to whether the data conformed to a normal distribution. The critical value of clinical significance was determined by the ROC curve Youden index according to the target status value (CAUTI or not) of age, days of hospitalization, days of indwelling urinary catheter and urine pH value, which were divided into two subgroups. An independent sample t test was used to compare the means of normally distributed variables, and the Wilcoxon rank sum test was used to compare the means of nonnormally distributed variables. The rate was compared by the chi-square test. Univariable and multivariable logistic regression models were used to analyse the associations between risk factors and CAUTI. Odds ratios (OR) with 95% confidence intervals (CI) were estimated. *P* < 0.05 was considered to be statistically significant.

## Results

### Risk factors for patients with CAUTI

A total of 7295 patients with indwelling urinary catheters were included in our study. The catheter was used for 53,523 days. There were 3926 males and 3369 females, aged 60–102 years, with a median age of 68 (64,75) years. Among the 7295 subjects, there were 7113 cases in the non-CAUTI group and 182 cases in the CAUTI group, and the infection rate was 3.4/1000 catheter days.

In addition, we further analysed the risk factors for CAUTI. Univariate logistic regression analysis showed that all variables except for sex (*p =* 0.994) had statistical significance (*p* < 0.001) (Table 1). Moreover, after all variables were analysed by regression model, the results showed that urine pH ≥ 6.5 (*OR* = 24.292, *95% CI* 15.736~37.499), moderate dependence(*OR* = 6.198, *95% CI* 2.681~14.328) or severe dependence(*OR* = 5.985, *95% CI* 2.848~12.579) in the classification of self-care ability, age ≥ 74 years old*OR* = 1.514, *95% CI* 1.036~2.212, male*OR* = 1.483, *95% CI*1.031~2.135, hospitalization ≥ 14 days*OR* = 7.500, *95% CI*4.408~12.762, indwelling urinary catheter ≥ 10 days(*OR* = 6.352, *95% CI* 3.941~10.236), diabetes*OR* = 2.602, *95% CI*1.748~3.871, malnutrition*OR* = 2.718, *95% CI*1.829~4.040were independent risk factors for CAUTI (*p*<0.05)(Table 2).

**Table 1:** Univariable logistic analysis of the risk factors for patients with CAUTI

Factor	CAUTI(n,%) n = 182	Non- CAUTI(n,%) n = 7113	*OR*	*95% CI*	*P*
**Age (years)**			2.319	1.726~3.115	<0.001
<74	95(52.20)	5099(71.69)			
≥ 74	87(47.80)	2014(28.31)			
**Gender**			0.999	0.744~1.342	0.994
Male	98(53.80)	3828(53.82)			
Female	84(46.20)	3285(46.18)			
**Check into the department**			3.067	2.281~4.124	<0.001
General ward	96(52.70)	5505(77.39)			
ICU	86(47.30)	1608(22.61)			
**Hospitalization (days)**			16.168	10.232~25.546	<0.001
<14	21(11.54)	4825(67.83)			
≥ 14	161(88.46)	2288(32.17)			
**Indwelling urinary catheter (days)**			22.982	15.613~33.831	<0.001
<10	32(17.58)	5908(83.06)			
≥ 10	150(82.42)	1205(16.94)			
**Urine pH**			9.283	6.491~13.277	<0.001
<6.5	39(21.43)	5099(71.69)			
≥ 6.5	143(78.57)	2014(28.31)			
**Fecal incontinence**			4.009	2.866~5.609	<0.001
No	132(72.53)	6499(91.37)			
Yes	50(27.47)	614(8.63)			
**State of consciousness**					
Waking	86(47.25)	6071(85.35)			<0.001
Somnolence	34(18.68)	486(6.83)	4.939	3.285~7.424	<0.001
Stupor	19(10.44)	142(2.00)	9.446	5.593~15.951	<0.001
Coma	36(19.78)	376(5.29)	6.759	4.518~10.111	<0.001
Dementia	7(3.85)	38(0.53)	13.004	5.649~29.935	<0.001
**Classification of self-care ability**					
No dependence	9(4.95)	2799(39.35)			<0.001
Mild dependence	12(6.59)	1751(24.62)	2.131	0.896~5.069	0.087
Moderate dependence	26(14.29)	780(10.97)	10.367	4.838~22.215	<0.001
Severe dependence	135(74.18)	1783(25.07)	23.547	11.961~46.357	<0.001
**Diabetes**			2.182	1.604~2.968	<0.001
No	116(63.74)	5642(79.32)			
Yes	66(36.26)	1471(20.68)			
**Malnutrition**			6.256	4.643~8.43	<0.001
No	90(49.45)	6114(85.96)			
Yes	92(50.55)	999(14.04)			
**Stroke**			1.807	1.314~2.486	<0.001
No	125(68.68)	5680(79.85)			
Yes	57(31.32)	1433(20.15)			

**Table 2:** Multivariable logistic analysis of the risk factors for patients with CAUTI

Factor	*OR*	*95% CI*	*P*
Urine pH ≥ 6.5	24.292	15.736~37.499	<0.001
Self-care ability classification			
No dependence			<0.001
Mild dependence	1.526	0.620~3.757	0.358
Moderate dependence	6.198	2.681~14.328	<0.001
Severe dependence	5.985	2.848~12.579	<0.001
Age ≥ 74 years	1.514	1.036~2.212	0.032
Male	1.483	1.031~2.135	0.034
Hospitalization ≥ 14 days	7.500	4.408~12.762	<0.001
Indwelling urinary catheter ≥ 10 days	6.352	3.941~10.236	<0.001
Diabetes	2.602	1.748~3.871	<0.001
Malnutrition	2.718	1.829~4.040	<0.001

### Distribution of opportunistic pathogens detected in urine samples of patients with CAUTI

A total of 276 strains of pathogens were detected in urine samples of 182 CAUTI patients at different times during hospitalization, mainly gram-negative bacteria (n = 132, 47.83%), followed by gram-positive bacteria (n = 91, 32.97%) and fungi (n = 53, 19.20%). The most common pathogens included *Enterococcus faecium*(*E.faecium*) (n = 73, 26.45%), *E.coli*(n = 44, 15.94%), *K. pneumoniae* (n = 40, 14.49%) and *Candida albicans*(*C.albicans*) (n = 28, 10.14%) .More than one pathogen was detected in 70 patients. (Table 3).

**Table 3:** Distribution and constituent ratio of opportunistic pathogens in urine samples of patients with CAUTI

Opportunistic pathogen	Number of strains	Constituent ratio (%)
**Gram-positive bacteria**		
*Enterococcus faecium*	73	26.45
*Enterococcus faecalis*	12	4.35
*Staphylococcus aureus*	3	1.09
*Corynebacterium Urealyticum*	1	0.36
*Enterococcus gallinarum*	1	0.36
*Enterococcus casseliflavus*	1	0.36
*Corynebacterium striatum*	1	0.36
**Gram-negative bacteria**		
*Escherichia coli*	44	15.94
*Klebsiella pneumoniae*	40	14.49
*Proteus mirabilis*	12	4.35
*Pseudomonas aeruginosa*	12	4.35
*Acinetobacter baumannii*	7	2.54
*Enterobacter cloacae*	6	2.17
*Proteus penneri*	3	1.09
*Stenotrophomonas maltophilia*	2	0.72
*Citrobacter freundii*	2	0.72
*Klebsiella oxytoca*	1	0.36
*Morganella morganii*	1	0.36
*Klebsiella aerogenes*	1	0.36
**Fungi**		
*Candida albicans*	28	10.14
*Candida tropicalis*	10	3.62
*Candida glabrata*	7	2.54
*Candida parapsilosis*	4	1.45
*Trichosporon Asahii*	3	1.09
*Candida lusitanias*	1	0.36
**Total**	276	100.00

### History of pre-infection antimicrobial therapy in patients with *E.coli* and *E.faecium*

The average number of days that patients with *E.coli* received antimicrobial therapy prior to infection diagnosis was 0 (0, 8) days, 11.5 (7.5, 23) days for *E.faecium* patients, and 18 (9,26) days for patients infected with both pathogens. There were 31 patients with *E.coli* infection, of whom 16 (51.64%) had not received antimicrobial therapy before infection, 60 patients with *E.faecium* infection, of whom 57 (95.00%) had received antimicrobial therapy before infection, and 13 patients with both pathogens had received antimicrobial therapy before infection(*p*<0.001)(Table 4).We also found that the antimicrobial exposure history of patients infected with *E.faecium* in this group was mainly the third-generation cephalosporins and carbapenems(Table 5).

**Table 4:** Comparison of antimicrobial therapy in patients infected with *E.coli* and *E.faecium*

Factor	*E.coli* n = 31	*E.faecium* n = 60	*E.coli* and *E.faecium* n = 13	*Z*/*x*^*2*^	*p*
Antimicrobial therapy days	0(0, 8)	11.5(7.5, 23)	18(9, 26)	16.604	<0.001
Antimicrobial therapy types(n,%)				31.664	<0.001^a^
No	16(51.61)	3(5.00)	0		
1 type	13(41.94)	40(66.67)	8(61.54)		
2 types	2(6.45)	14(23.33)	5(38.46)		
3 types	0	3(5.00)	0		

^a^ Fisher’s exact test

**Table 5:** List of antimicrobial exposure in patients infected with *E.coli* and *E.faecium*

Antimicrobial	*E.coli* (n,%)	*E.faecium* (n,%)	*E.coli* and *E.faecium* (n,%)
Cefperazone-sulbactam	5(2.72)	34(18.48)	5(2.72)
Cefotaxime-sulbactam	1(0.54)	11(5.98)	1(0.54)
Ceftazidime	1(0.54)	7(3.8)	2(1.09)
Ceftriaxone	3(1.63)	6(3.26)	0
Cefperazone-tazobactam	0	2(1.09)	0
Cefuroxime	1(0.54)	0	0
Meropenem	0	20(10.87)	3(1.63)
Imipenem-cilastatin	1(0.54)	4(2.17)	2(1.09)
Biapenem	0	3(1.63)	2(1.09)
Gentamicin	1(0.54)	0	1(0.54)
Amikacin	0	0	1(0.54)
Moxifloxacin	4(2.17)	6(3.26)	1(0.54)
Levofloxacin	2(1.09)	8(4.35)	2(1.09)
Piperacillin—tazobactam	2(1.09)	12(6.52)	3(1.63)
Linezolid	2(1.09)	2(1.09)	0
Vancomycin	2(1.09)	1(0.54)	0
Tigecycline	1(0.54)	5(2.72)	0
Clindamycin	0	1(0.54)	0
Rifampin	0	1(0.54)	0
Metronidazole	0	0	1(0.54)
Ornidazole	0	2(1.09)	0
Fluconazole	0	3(1.63)	2(1.09)
Voriconazole	0	2(1.09)	1(0.54)
Caspofungin	0	1(0.54)	0
Total	26(14.13)	131(71.20)	27(14.67)

### Analysis of clinical characteristics of patients with CAUTI

The subjects were divided into two groups according to CAUTI and non-CAUTI status. The results showed that the proportions of patients with fever, abnormal procalcitonin, positive urinary nitrite and abnormal urination function were higher than those in the non-CAUTI group (*p* < 0.001) (Table 6).

**Table 6:** Comparison of clinical data between the two groups

Characteristic	CAUTI(n,%) n = 182	Non- CAUTI(n,%) n = 7113	*x* ^*2*^	*p*
**Fever**			180.608	<0.001
No	55(30.22)	4989(70.14)		
1 day	27(14.84)	899(12.64)		
≥ 2 days	100(54.95)	1225(17.22)		
**Procalcitonin**			107.262	<0.001^a^
Normal	30(16.48)	3806(53.51)		
Abnormal	152(83.52)	3189(44.83)		
Not detected	0	118(1.66)		
**Urinary nitrite**			22.985	<0.001
Negative	155(85.16)	6680(93.91)		
Positive	27(14.84)	433(6.09)		
**Urination function**			247.542	<0.001
Normal	44(24.18)	5385(75.71)		
Abnormal	138(75.82)	1728(24.29)		

^a^ Fisher’s exact test

### Outcomes of patients with CAUTI

Finally, we analysed the factors affecting the prognosis of patients with CAUTI. Compared with the non-CAUTI group, the hospitalization days of the CAUTI group increased by 18 days (10 vs. 28 days), the total hospitalization cost increased by ¥18,000 (89,000 vs. 107,000 yuan), and discharge all-cause mortality increased by 2.314 times (*OR = 3.314, 95% CI* 2.002~5.488) (*p*<0.001) (Table 7).

**Table 7:** Outcomes between the two groups

Outcome	CAUTI n = 182	Non-CAUTI n = 7113	*Z*	*P*
Hospitalization(days)	28(16, 48)	10(7, 15)	-17.216	<0.001
Total cost of hospitalization (10,000 yuan)	10.70(5.89, 22.76)	8.90(5.54, 14.03)	-4.414	<0.001
Discharge all-cause mortality(n,%)			/	<0.001^a^
No	164(90.11)	6885(96.79)		
Yes	18(9.89)	228(3.21)		

^a^*OR* = 3.314, *95% CI* 2.002~5.488

## Discussion

Our study divided the urine pH value into two subgroups based on the critical value judged by the ROC curve Youden index. The results showed that a urine pH value ≥ 6.5 was an independent risk factor for CAUTI. The median urine pH value in the CAUTI group was 7 (6.5, 7.5) and that in the non-CAUTI group was 6 (5.5, 6.5). This is rare in previous studies. We chose the timing of the urine pH study at the initial admission stage and before the indwelling catheter. The main reason is to reduce the research bias of urine results caused by disease treatment and invasive operation. It is well known that an acidic environment is not conducive to bacterial growth [13], and acidic urine also has a certain protective effect on CAUTI, as confirmed in our study. In clinical practice, we can use this conclusion as a guide through high-safety intervention methods to maintain the urinary environment of patients with catheterization in the normal range and pH < 6.5, which is conducive to the prevention of CAUTI.

We found that moderate dependence or severe dependence in the classification of self-care ability was an independent risk factor for CAUTI. The self-care ability grade in this study is that patients are graded by nurses in charge of the Barthel index rating scale at admission, which can truly reflect the patients’ self-care ability. The self-care ability score scale mainly measures the patients’ ability to eat independently, take a bath, go to the toilet, walk and so on. Many previous studies have shown that cerebrovascular disease, paraplegia or dyskinesia are risk factors for CAUTI [4, 14, 15]. This kind of patient has an increased incidence of CAUTI due to the limitation of activity caused by disease, which is consistent with the conclusion of this study.

In addition, our study shows that males are more prone to CAUTI than females, which is different from previous studies [15]. Advanced age, long hospital stay, long indwelling urinary catheter and diabetes have been unanimously recognized as risk factors for CAUTI in many previous studies [16–18], which is no exception in our study.However, our research has clarified the time characteristics of CAUTI in elderly individuals.

Malnutrition in the elderly has been shown to be associated with an increased risk of hospitalization and death [19]. In our study, it was shown that it is an independent risk factor for CAUTI, suggesting that nutritional status assessment is a very important issue in the process of diagnosis and treatment. Improving the nutritional status of elderly individuals is one of the strategies to prevent and control infection, and early intervention should be carried out.

In our study, 276 strains of opportunistic pathogens were detected in 182 cases of CAUTI. The distribution of pathogens was mainly gram-negative bacteria, followed by gram-positive bacteria and fungi. The primary pathogen causing CAUTI is *E.faecium*, followed by *E.coli, K.pneumoniae* and *C.albicans*, which differs from some studies [20–22]. This is mainly because *E.faecium* and *E.coli* are usually parasitic in the human intestinal flora and elderly individuals due to physical function decline, low immunity, many primary diseases and other reasons. This provides an opportunity for bacterial invasion. It is inferred that the occurrence of CAUTI in elderly individuals is closely related to endogenous infection. At the same time, *E.faecium* was the most common pathogen in this study, which was different from previous reports. We reviewed the history of antimicrobial exposure during hospitalization in this patient population from the first day of admission until the first diagnosis of CAUTI caused by *E. coli* or *E. faecium*.We found that patients infected only with *E.faecium* and with both pathogens had a high probability of receiving antimicrobial therapy, and the time was longer than that of patients infected only with *E.coli.*There were 31 patients infected only with *E.coli*, 16 of whom had not received antimicrobial therapy before infection, and 60 patients infected only with *E.faecium*, 57 of whom had received antimicrobial therapy before infection.The high incidence of *E.faecium* is related to the history of antimicrobial exposure.We also found that the antimicrobial exposure history of patients infected with *E.faecium* in this group was mainly third-generation cephalosporins and carbapenems,while *Enterococcus* was naturally resistant to cephalosporins.When *E.coli* and *E. faecium* are both opportunistic intestinal parasitic pathogens, the tendency of clinicians in this hospital to use drugs leads to *E.faecium* becoming the dominant pathogen, with a higher incidence than *E.coli.*In addition, the incidence of *C.albicans* infection is not low, accounting for 10.14%, which is different from the distribution of UTI pathogens in nonelderly patients [23]. It is considered that it is mainly related to many kinds of pathogens in the elderly and the extensive use of broad-spectrum antibiotics. Thus, the treatment of CAUTI in elderly patients should strictly abide by etiological examination before the use of antibiotics to avoid multidrug-resistant organism infections or complex infections caused by the unreasonable use of antibiotics. At the same time, our study provides a reference for the empirical application of antibiotics before etiological examination results.

In addition, we found that 70 of the 182 infected patients had more than one opportunistic pathogen during hospitalization (including 45 had two, 18 had three, and 7 had four or more). A total of 164 pathogens were detected in 70 patients, mainly *E.faecium* (25.61%), *K.pneumoniae* (17.68%), *E.coli* (14.02%), *C.albicans* (11.59%) and *P.aeruginosa* (6.71%).Analysis of the patients showed that 53 of the 70 patients with multi-pathogen infection were admitted to the geriatrics, neurology and rehabilitation departments for chronic diseases, and the length of hospitalization was generally long, with a minimum of 7 days and a maximum of 436 days, and the average hospitalization was 37.5 (22,61) days (median). In conclusion, a long hospital stay is the main risk factor for multi-pathogen infection in elderly patients.

Our study was divided into two groups, the CAUTI group and the non-CAUTI group, to investigate the clinical characteristics of elderly patients with indwelling urinary catheters. The results showed that fever was the clinical feature of elderly CAUTI patients, and 70% of the infected people had fever symptoms. It is well known that fever is a nonspecific symptom of infection. The judgment of CAUTI should be combined with clinical symptoms and signs in addition to indwelling urinary catheter time and urine culture results. Patients with indwelling urinary catheters have no obvious feeling of urinary symptoms, such as frequent urination and urgent urination, and most elderly inpatients have varying degrees of unconsciousness, increasing the difficulty of determining CAUTI. If the judgment is wrong, the optimal time for treatment may be missed. Therefore, in clinical diagnosis and treatment, when elderly patients with indwelling urinary catheters have fever symptoms, attention should be given to the identification of other infections that cannot be excluded from CAUTI determination because they are clinically deemed due to another recognized cause.

In addition, the detection of procalcitonin and urinary nitrite is also an auxiliary index commonly used in the diagnosis of CAUTI. Our analysis results show that compared with the group without CAUTI, these two tests are meaningful, but they have more advantages in determining procalcitonin in infection, with an abnormal rate of 83.52%, which can be considered one of the effective indicators for the diagnosis of CAUTI in elderly individuals.

We found that older patients with urinary retention or incontinence were more likely to develop CAUTI than those with normal urination function. Considering the high incidence of urinary retention and incontinence in the elderly [24, 25], it is the main indication for indwelling urinary catheters in elderly patients, and it requires a long duration of indwelling. The degeneration of urinary tract mucosa and the decrease in local antibacterial ability in elderly individuals may increase the risk of CAUTI. Therefore, elderly patients with abnormal urination function should be taken as the key population for infection prevention and control.

Finally, we analysed the prognosis of CAUTI. We found that CAUTI could significantly affect the clinical outcome of patients, including prolonged hospitalization, increased hospitalization costs and increased all-cause mortality, which was consistent with related research conclusions [26, 27]. In recent years, there have been many studies on CAUTI, but few studies have clarified the impact of adverse outcomes based on a large sample size. The results of this study provide a useful supplement to this. Our study showed that compared with the non-CAUTI group, the hospitalization days of the CAUTI group increased by 18 days, the total hospitalization cost increased by ¥18,000, and discharge all-cause mortality increased by 2.314 times. To save national medical resources, improve patient safety and reduce mortality, clinical doctors and nurses should pay more attention to the prevention, control and management of CAUTI. In daily work, guiding suggestions and prevention and control measures in national norms and expert consensus should be implemented.

There are several limitations to this study. First, our study was conducted in a single hospital, which limits the representativeness and extrapolation of results from a single center. Second, our study is a retrospective analysis with limited data, but it points out the direction for our future research. We will carry out a prospective study on this subject.

## Conclusion

The situation of CAUTI in the elderly is not optimistic, it is easy to have one-person multi-pathogen infection, and the proportion of fungal infection is not low. Our study uses a large sample size to determine the risk factors different from other studies, providing a new idea for the prevention and control of aged CAUTI. Meanwhile, the clinical characteristics of aged CAUTI patients were analysed and summarized. It provides a reliable basis for its diagnosis and clarifies the adverse outcomes of CAUTI. These findings are important for preventing and controlling CAUTI in aged individuals.

## Data Availability

The datasets used and analysed during the current study are available from the corresponding author on reasonable request.

## Abbreviations

CAUTI: Catheter-associated Urinary Tract Infection
UTI: Urinary Tract Infection
ATCC: American Type Culture Collection
SPSS: Statistical Package for Social Science
CDC: Center for Disease Control
NHSN: National Healthcare Safety Network
HIS: Hospital Information System
LIS: Laboratory Information System
